# Recent updates on amino acid requirements of pigs based on phenotypic improvements: invited review

**DOI:** 10.5713/ab.250891

**Published:** 2026-02-06

**Authors:** Hyunjun Choi, Jung Yeol Sung, Jeonghyeon Son, Sung Woo Kim

**Affiliations:** 1 Department of Animal Science, North Carolina State University, Raleigh, NC, USA

**Keywords:** Amino Acids, Growth Performance, Lysine, Meta-analysis, Pigs, Requirement

## Abstract

Amino acid (AA) requirements reported in previous literature, such as those of the National Research Council (NRC) published in 2012 based on pre-2010 data, do not adequately reflect the improved genetic potential of pigs and thus require updating. Accordingly, this review aimed to provide recent updates on the AA requirements of pigs based on the standardized ileal digestible (SID) lysine (Lys) to net energy (NE) ratio (SID Lys:NE) and the ideal ratios of SID methionine (Met), threonine (Thr), tryptophan (Trp), valine (Val), and isoleucine (Ile) to SID Lys (SID AA:Lys), derived from meta-analyses for average daily gain (ADG) and gain-to-feed ratio. The optimal SID Lys:NE for ADG in this review are consistently greater than the ratios reported by the NRC across different phases, indicating that the improved genetic potential for lean growth requires greater optimal SID Lys:NE. The optimal SID Met:Lys, SID Trp:Lys, and SID Ile:Lys for ADG remain constant with increasing body weight (BW) of pigs, but the ratios are also greater than those reported by the NRC. However, the optimal SID Thr:Lys for ADG increases as BW of pigs increases, likely due to the increasing proportion of maintenance requirement relative to the total Thr requirement. The optimal SID Val:Lys for ADG is greater in the nursery phase than in the grower phases, suggesting that weaning-associated intestinal and immune challenges would increase requirement for Val. Both optimal SID Thr:Lys and SID Val:Lys are greater than the ratios reported by the NRC. In conclusion, these results suggest that the optimal SID Lys:NE and SID AA:Lys in pig diets are greater than the ratios reported by the NRC, and different ratios of SID Thr:Lys and SID Val:Lys should be applied for pigs with different BW.

## INTRODUCTION

**Figure 1:**
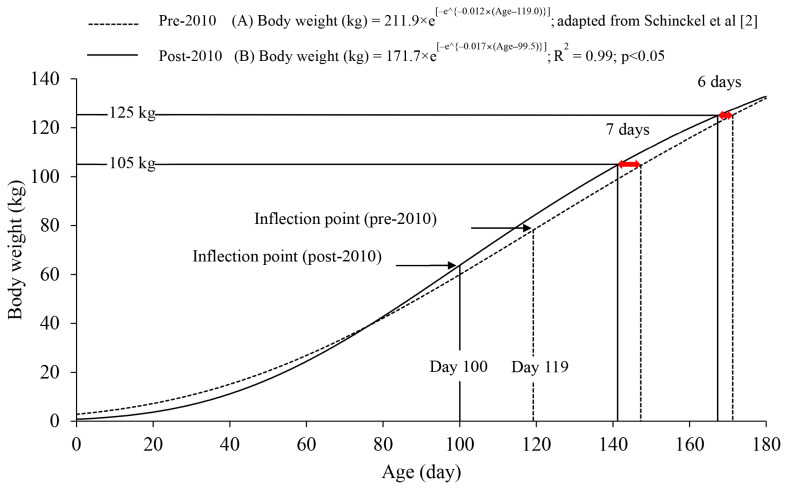
Body weight as a function of age in mixed-sex pigs modeled using the Gompertz equation, based on data published (A) pre-2010 [2] and (B) post-2010 [3–29] in the United States. The inflection points of the body weight curves, based on data published pre-2010 (dashed line) and post-2010 (solid line), were at days 119 and 100, respectively. Compared with data published pre-2010, pigs post-2010 reached 105 and 125 kg 7 and 6 days earlier, respectively.

The genetic potential for lean growth of pigs has improved, as demonstrated by increased growth rate compared with pigs of the past [1]. A growth curve generated by Schinckel et al [2] based on data from 2003 to 2005 (pre-2010) indicates that pigs reached 125 kg at 173 days of age, whereas a growth curve generated using post-2010 data [3–29] indicates that pigs with improved genetic lines reached 125 kg approximately 6 days earlier (Figure 1). This improvement is due to a shift in the inflection point from day 119 to 100 based on pre-2010 and post-2010 growth curves, respectively. The shift in the inflection point indicates that pigs with improved genetic lines reach their maximum growth rate earlier in life, reflecting genetic selection for pigs that reach target weights more rapidly than pigs of the past [30]. Considering the improved growth potential of pigs, previous dietary amino acid (AA) requirements, such as those in the NRC [31] based on pre-2010 data, may not meet the requirements of AA of pigs with improved genetic potential and thus need to be updated.

AA requirements are generally expressed on a standardized ileal digestible (SID) basis [32,33], which excludes the impact of microbial fermentation of AAs in the large intestine and accounts for basal endogenous AA losses, thereby providing accurate and reliable estimates. The SID lysine (Lys) to net energy (NE) ratio (SID Lys:NE) is used to express Lys requirements because dietary NE is a major factor regulating feed intake [34,35]. Because Lys is the first-limiting AA in cereal grain- and soybean meal-based diets and the most abundant AA in muscle [36], its inclusion in the diet relative to energy intake must be considered. As a result, when expressing the requirements of the other AAs, they are typically expressed relative to Lys [37–39] as an ideal protein ratio (SID AA:Lys). Expressing AA requirements relative to Lys also directly reflects the balance of AAs for protein synthesis, thereby preventing both deficiencies and excesses and ensuring optimal utilization of dietary protein and AAs. Thus, the SID Lys:NE and SID AA:Lys have been used to express AA requirements in pig nutrition.

**Figure 2:**
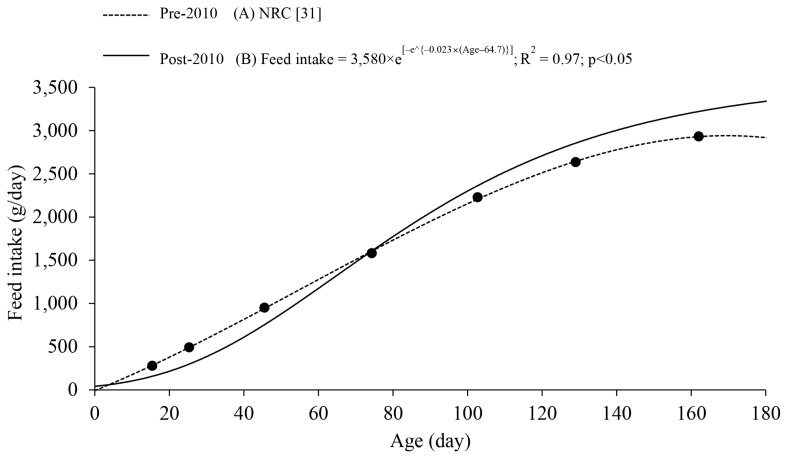
Feed intake as a function of age in mixed-sex pigs using (A) the NRC [31] data based on pre-2010 (dashed line) and (B) the Gompertz equation based on data published in the United States post-2010 (solid line) [3–11,15–18,20–29]. Solid dots represent estimated feed intake of pigs with different body weight range provided by the NRC [31].

AA requirements can be expressed either as daily intake (g/d) or as a percentage of the diet (%). When formulating pig diets, AA requirements are expressed as a percentage of the diet (%) and are calculated by dividing daily AA requirements (g/d) by daily feed intake (g/d) of pigs. AA requirements expressed as daily intake (g/d) will gradually increase as body weight (BW) of pigs increases [31]. However, AA requirements expressed as a percentage of the diet (%) gradually decrease as BW increases because feed intake (g/d) increases at a faster rate than daily AA requirements (g/d; Figure 2). As pigs grow, the proportion of fat to lean tissue in the body increases [40,41]. Therefore, dietary AA concentration in pig diets should be adjusted according to BW. However, continuous adjustment of dietary AA concentration is not feasible and thus a gradual adjustment called ‘phase feeding’ has widely been applied in practice. Based on the background, this review aimed to update the AA requirements at different phases, expressed as the optimal SID Lys:NE and the ideal ratios of SID methionine (Met), threonine (Thr), tryptophan (Trp), valine (Val), and isoleucine (Ile) to SID Lys for average daily gain (ADG) and gain-to-feed ratio (G:F) using meta-analyses of 327 experiments (118, 40, 50, 71, 29, and 19 for Lys, Met, Thr, Trp, Val, and Ile, respectively).

## THE REQUIREMENTS FOR STANDARDIZED ILEAL DIGESTIBLE LYSINE:NET ENERGY AND THE IDEAL PROTEIN RATIO (STANDARDIZED ILEAL DIGESTIBLE AMINO ACID:LYSINE)

Accurate determination of AA requirements in pigs is essential for optimizing protein synthesis, growth performance, and nutrient utilization. Considering that the majority of AA absorption is completed prior to the end of the small intestine [37] and AA profile of digesta would be altered from microbial fermentation in the large intestine [42,43], AA requirements for pigs are expressed on ileal digestible AA basis [42,44,45] rather than on total tract digestible AA basis. Ileal digestible AA can be further classified on an apparent, true, or standardized basis. Apparent ileal digestible AA should not be used to express AA requirements as it does not consider total endogenous AA losses, leading to an underestimation of digestible AA in mixed diets [32,46]. The concept of true ileal digestible AA was introduced to address this limitation by considering total endogenous AA losses, which consist of specific and basal endogenous AA losses. However, the use of true ileal digestible AA is limited because the levels of specific endogenous AA losses vary depending on the feedstuffs. Furthermore, measuring specific endogenous AA losses is costly and requires labor-intensive methods, such as isotope dilution or homoarginine technique [47,48]. Therefore, using the SID AA basis corrects apparent ileal digestible AA values only for basal endogenous AA losses, which can be measured by using a nitrogen-free diet or a diet containing highly digestible protein supplement such as casein [44,49,50]. Thus, the SID AA basis has been widely used to express AA requirements in pig nutrition. Dietary energy plays a critical role in determining AA requirements. Because voluntary feed intake is regulated by energy intake [51,52], the AA intake is dependent on dietary energy content. Among energy systems, the NE system provides a more accurate estimate of available energy compared with digestible energy and metabolizable energy (ME) [53–55]. This is because the NE system accounts for metabolic heat, the energy lost as heat during digestion and metabolism, and thus provides reliable values regardless of changes in the types of feedstuffs used in diets [53,54]. Lys is the first-limiting AA in pigs fed cereal grain- and soybean meal-based diets and plays a crucial role in protein synthesis and growth. Thus, the requirements of other AAs are usually expressed as a ratio relative to Lys [36]. Accordingly, the SID Lys:NE and SID AA:Lys for pigs are widely used to express the AA requirements.

Growing interests and needs of animal agriculture for environmental sustainability have led animal nutritionists to proactively reduce crude protein contents in diets, whilst ensuring adequate essential AAs supplementation, so called low-protein formulation. Feeding low-protein diets could reduce the metabolic heat, feed costs, and nitrogen excretion [56–59]. Accurate estimation of AA requirements becomes particularly important in the context of low-protein formulation because inaccurate estimates would lead to AA imbalance and deficiencies compared with conventional formulation. Therefore, the precise estimation of SID Lys:NE and SID AA:Lys has become increasingly important in sustainable pig production.

## PHASE FEEDING

Phase feeding is one of the practical feeding strategies in which diets are divided into multiple phases according to BW range to provide adequate amounts of AA [31]. As stated above, AA requirements can be expressed either as daily intake (g/d) or as a percentage of the diet (%). In diet formulation, AA requirements are generally expressed as a percentage of the diet (%), which is estimated by dividing required daily AA intake (g/d) by daily feed intake (g/d) of pigs. As pigs grow, the increase of voluntary feed intake is greater than the increase of daily AA requirements (g/d) resulting in gradual decrease of required dietary AA concentration (%) [31]. The proportion of fat to lean tissue in the body changes as pigs grow [40,41]. In this context, continuous adjustment of dietary AA contents according to BW is theoretically ideal from the perspective of precision nutrition, as it allows the AA requirements of pigs at each BW to be met, thereby improving growth performance and reducing nitrogen excretion [60,61]. Despite its theoretical rationale, continuous adjustment of dietary AA contents according to BW is not feasible due to complexity of feed delivery and practice of group feeding. Therefore, a gradual adjustment called ‘phase feeding’ is commonly applied in practice [62,63]. In accordance with phase feeding concept, the NRC [31] provides an example of seven feeding phases based on BW ranges for growing pigs (5 to 7 kg, 7 to 11 kg, 11 to 25 kg, 25 to 50 kg, 50 to 75 kg, 75 to 100 kg, and 100 to 135 kg).

## EMPIRICAL METHOD TO ESTIMATE AMINO ACID REQUIREMENTS

Two main methods are commonly used to estimate AA requirements: the empirical method and the factorial method [64]. AA requirements estimated by the empirical method represent the minimal amount of AA to optimize responses of population during a given time, whereas AA requirements estimated by the factorial method represent amount of AA required for an individual pig, combining the amounts needed for maintenance and production at a specified time point [65–67]. In this review, only the empirical method is discussed.

To estimate AA requirements by the empirical method, titration studies should be conducted in which pigs are tested at different levels of the AA of interest [31,51]. In titration studies, the basal diet should be formulated to contain the lowest level of the AA of interest among all diets, thereby ensuring that it is deficient and serves as the first-limiting AA [67]. Lys should be the second-limiting AA in the basal diet, whereas the supply of the other AAs, except for the AA of interest and Lys, should meet or slightly exceed the requirements to ensure that the other AAs do not become limiting [51,68]. The optimal SID AA:Lys is assumed to be unaffected by dietary Lys content [67]. The AA of interest is supplemented to the basal diet at graded levels, ranging from deficient to exceeding the estimated requirement.

**Figure 3:**
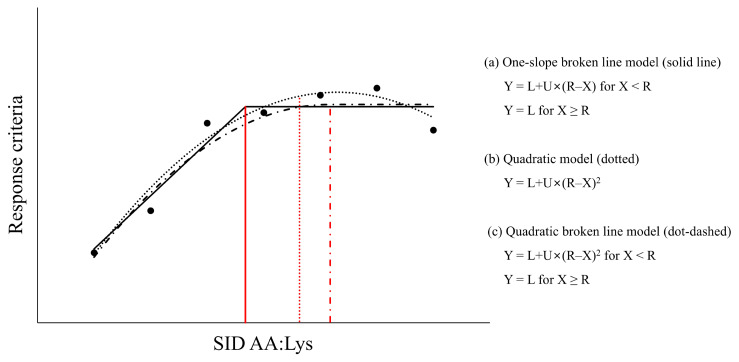
Example of standardized ileal digestible (SID) amino acids (AA) to SID lysine (SID AA:lysine [Lys]) using different models. (A) The one-slope broken line model is expressed as Y = L+U×(R–X) when X is less than R and Y = L when X reaches or exceeds R (solid lines). (B) The quadratic model is expressed as Y = L+U×(R–X)^2^ for all estimates of X (dotted lines). For the quadratic model, the requirement was defined as 95% of the maximum response. (C) The quadratic broken line model is expressed as Y = L+U×(R–X)^2^ when X is less than R and Y = L when X reaches or exceeds R (dot-dashed lines). L represents the response criteria such as growth performance at the requirement and U represents the slope (one-slope broken line model) or curvature coefficient (quadratic model or quadratic broken line model) of the response. In the one-slope and quadratic broken line analyses, R represents the break point that defines the requirement, whereas in the quadratic model, R corresponds to the point of maximum response.

To estimate the optimal SID Lys:NE and SID AA:Lys based on the empirical method, three statistical models were used in this review (Figure 3). As previously suggested by Robbins et al [69] and Pomar et al [66], AA requirements may vary depending on the models due to their underlying assumptions:

The one-slope broken line model assumes a linear change in the response variable (e.g., ADG or G:F) with increasing levels of the AA of interest until the requirement is met [66,69]. The breakpoint is considered the requirement.

The quadratic broken line model assumes a gradually reduced change in the response variable as the level of the AA of interest approaches the requirement, followed by a plateau beyond the breakpoint [66,69]. The breakpoint is considered the requirement. Typically, the quadratic broken line model may better represent the response of the population, and AA requirements estimated by the quadratic broken line model are greater than those estimated by the one-slope broken line model [66,70].

The quadratic model is also commonly used to estimate AA requirements and assumes a curvilinear response below the breakpoint, which may better reflect biological responses [51,69]. However, the response (e.g., growth performance) does not plateau with increasing AA levels in the quadratic model and may even decrease beyond the estimated requirement, which is not typically observed biologically [70]. AA requirements estimated by the quadratic model are typically greater than those estimated by one-slope broken line model or quadratic broken line model because estimated values in the quadratic model are obtained at the maximal growth of pigs [51,69]. Therefore, the AA requirements estimated by the quadratic model are typically adjusted to 95% of the maximal response to adjust its potential bias [68].

## DATA COLLECTION

To estimate accurate AA requirements (SID Lys:NE and SID AA:Lys) across multiple phases according to BW range, meta-analyses were conducted. Only the data published in peer-reviewed papers were used in the analyses. Peer-reviewed papers published from 1990 to 2025 were searched in PubMed, Scopus, and Google Scholar using the following keywords: AA (Lys, Met, Thr, Trp, Val, or Ile), requirement, pigs, and growth-related terms such as growth performance, growth, weight gain, gain-to-feed, feed conversion rate, or feed efficiency. Subsequently, the collected papers were screened and included in the meta-analyses only if they followed the conditions of the empirical method outlined previously in this review and reported at least 3 dietary levels of the AA of interest. Data pertaining to sows in the collected papers were excluded from this review. Moreover, the collected papers in which pigs were exposed to immunological challenges, such as enterotoxigenic *Escherichia coli* or lipopolysaccharide, were excluded to avoid the potential confounding impact of immune challenge on AA requirements in pigs [71,72]. Overall, 327 experiments, including 118 for Lys, 40 for Met, 50 for Thr, 71 for Trp, 29 for Val, and 19 for Ile, were used to estimate the optimal SID Lys:NE or SID AA:Lys in pigs. Some individual papers included multiple experiments.

When dietary SID Lys (%) and NE (kcal/kg) were reported in the collected papers, these values were used in the meta-analyses. If not reported, dietary SID Lys was calculated by multiplying the total dietary Lys content by the standardized ileal digestibility (SID) of Lys based on feedstuff composition tables in the NRC [31], whereas dietary NE (kcal/kg) was calculated by multiplying dietary ME by a coefficient of 0.74 [73]. Although NE-to-ME conversion coefficient depends on the diet composition [54,74], a coefficient of 0.74 was used because it is the most commonly applied to the diets in the collected papers used for meta-analysis [54,55]. ADG and G:F were used as the response criteria in this review. The growth performance data were divided into 7 multiple phases according to BW range (5 to 7 kg, 7 to 11 kg, 11 to 25 kg, 25 to 50 kg, 50 to 75 kg, 75 to 100 kg, and 100 to 135 kg) that are presented in the NRC [31]. In some experiments, BW range of the pigs reported in the collected papers did not correspond to a single phase, therefore, the phase corresponding to the average BW of the pigs was used for the analysis.

## STATISTICAL ANALYSES

Meta-analyses were conducted using the PROC NLMIXED of SAS (SAS Institute) to estimate the dietary AA requirements (SID Lys:NE or SID AA:Lys) using broken line analyses [67]. Three non-linear models were used: the one-slope broken line model, quadratic model, and quadratic broken line model [52,70]. In the quadratic model, 95% of the upper asymptotic value for the breakpoint was considered as the optimal dietary AA requirement [68]. The statistical regression models were as follows:

One-slope broken line model


(1) Y=L+U×(R-X)+i+ɛ for X<R (with [R-X] defined as zero when X≥R)

Quadratic model


(2) $$\text{Y}=\text{L}+\text{U}\times {\left(\text{R}-\text{X}\right)}^{2}+i+ɛ\;(\text{the optimal X was defined as the level at 95}\%\text{ of the upper asymptote)}$$

Quadratic broken line model


(3) $$\text{Y}=\text{L}+\text{U}\times {\left(\text{R}-\text{X}\right)}^{2}+i+ɛ\;\text{for X}<\text{R }(\text{with }[\text{R}-\text{X}]\;\text{defined as zero when X}\geq \text{R})$$

**Figure 4:**
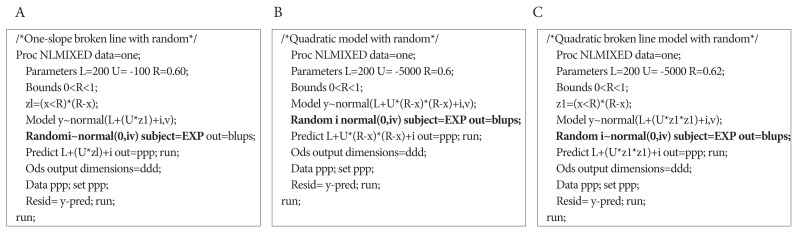
Examples of the SAS code used for the meta-analysis: (A) one-slope broken line model with random, (B) quadratic model with random, and (C) quadratic broken line model with random. In the meta-analysis using three models, individual experiments (EXP) were treated as a random effect (specified “i~normal(0,iv); subject=EXP”) to account for the inter-experiment variability.

In these models, Y is the dependent variable (ADG or G:F); L is the asymptote; U is the slope; X is the independent variable (SID Lys:NE or SID AA:Lys); R is the breakpoint, where (R–X) is defined as zero when X≥R; *i* is the random effect of experiment; and *ɛ* is the residual error. The fitted models were selected when p<0.05 for the asymptote, slope, and breakpoint. Individual experiments were considered as a random effect to account for the inter-experiment variability [58,75], as illustrated in Figure 4.

The prediction equations for optimal SID Lys:NE for ADG were established using the PROC NLIN of SAS by regressing optimal SID Lys:NE for ADG on mean BW of multiple phases. The statistical regression model was as follows:


(4) $$\text{Y}=\text{a}+\text{bX}+{\text{cX}}^{2}+{\text{dX}}^{3}+ɛ$$

In the model, Y is the dependent variable (SID Lys:NE, g/Mcal); X is the independent variable (mean BW of each phase, kg); a is the intercept; b is the linear coefficient; c is the quadratic coefficient; d is the cubic coefficient; and *ɛ* is the residual error. Statistical significance and tendency were declared at p<0.05 and 0.05≤p<0.10, respectively.

## OPTIMAL STANDARDIZED ILEAL DIGESTIBLE LYSINE TO NET ENERGY RATIO

Supplemental Lys is commonly added to pig diets to meet their Lys requirements as Lys is the first-limiting AA in pigs fed cereal grain- and soybean meal-based diets [31]. Lys plays a central role in protein synthesis, lean tissue accretion, and muscle development, and it is abundantly present in body proteins, particularly in muscle [36]. Approximately 19 to 20 g of SID Lys is required per kg of BW gain, which is the highest among all essential AAs [31]. Lys serves as a building block for proteins and supports immune function by enhancing antibody production and immune cell activity [72].

Meta-analyses of 118 experiments were conducted to estimate the optimal SID Lys:NE for ADG and G:F across multiple phases according to BW range (Table 1). The optimal SID Lys:NE for ADG decreases as BW of pigs increases from 7 to 135 kg, ranging from 5.2 to 2.6 g/Mcal in the one-slope broken line model, 5.8 to 2.8 g/Mcal in the quadratic model, and 5.9 to 2.9 g/Mcal in the quadratic broken line model (Table 2). The optimal SID Lys:NE for G:F decreases as BW of pigs increases, ranging from 5.4 to 2.3 g/Mcal in the one-slope broken line model from 7 to 135 kg, 6.3 to 2.7 g/Mcal in the quadratic model from 11 to 135 kg, and 6.7 to 2.7 g/Mcal in the quadratic broken line model from 11 to 135 kg.

The decrease in SID Lys:NE with increasing BW of pigs is partially explained by the relatively lower Lys requirement (expressed as percentage of the diet) and the greater fat deposition in growing-finishing pigs compared with nursery pigs. Nursery pigs face challenges associated with weaning stress, including reduced feed intake, tissue catabolism, and immunological challenges [76]. Lys is essential for nursery pigs to mitigate weaning stress by supporting muscle growth and immune functions, resulting in a greater SID Lys:NE for nursery pigs compared with growing-finishing pigs [31]. Moreover, the rate of protein deposition relative to energy intake decreases in growing-finishing pigs compared with nursery pigs because whole-body protein deposition reaches its maximum level at 60 to 80 kg of BW and then begins to decline [31]. In contrast to protein deposition, the rate of fat deposition relative to energy intake gradually increases in growing-finishing pigs and is further accelerated by increased energy intake as pigs grow [40,41].

**Figure 5:**
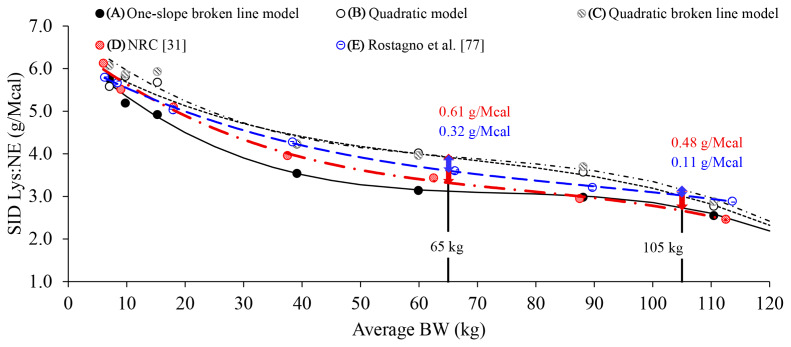
Comparison of the optimal standardized ileal digestible (SID) lysine (Lys):net energy (NE) ratio from this review and with those reported in the literature across the body weight (BW) range of pigs from weaning to market weight. (A) Prediction equation for the optimal SID Lys:NE ratio based on average daily gain (ADG) of pigs using a one-slope broken line model was: SID Lys:NE (g/Mcal) = −0.0000076×BW^3^+0.0017× BW^2^–0.130×BW+6.48; R^2^ = 1.00; p<0.01 (solid lines). (B) Prediction equation for the optimal SID Lys:NE ratio based on ADG of pigs using a quadratic model was: SID Lys:NE (g/Mcal) = −0.0000049×BW^3^+0.0010×BW^2^–0.082×BW+6.40; R^2^ = 0.97; p<0.01 (dotted lines). For the quadratic model, the requirement was defined as 95% of the maximum response. (C) Prediction equation for optimal SID Lys:NE ratio based on ADG of pigs using a quadratic broken-line model was: SID Lys:NE (g/Mcal) = −0.0000070×BW^3^+0.0014×BW^2^–0.110×BW+6.92; R^2^ = 0.98; p<0.01 (dot-dashed lines). (D) Optimal SID Lys:NE ratio from NRC [31] across the BW range of pigs (red long dash dot). (E) Optimal SID Lys:NE ratio from Rostagno et al [77] across the BW range of pigs (blue long dash). The differences between the optimal SID Lys:NE ratio from this review using the quadratic broken line model (C) and the NRC [31] (D) at 65 kg (inflection point) and 105 kg (where pre-2010 and post-2010 growth curves differed by 7 days) were 0.61 g/Mcal and 0.48 g/Mcal (red double-headed arrow), indicating that the previous recommendation may need to be updated. The differences between the optimal SID Lys:NE ratio from this review using the quadratic broken line model (C) and Rostagno et al [77] (E) at 65 kg and 105 kg were 0.32 g/Mcal and 0.11 g/Mcal (blue double-headed arrow).

The optimal SID Lys:NE estimated for ADG (5.8 to 2.8 g/Mcal from 7 to 135 kg) in this review, as estimated using both the quadratic model and quadratic broken line model, were consistently greater than the ratios reported by the NRC [31] (5.5 to 2.5 g/Mcal from 7 to 135 kg). In particular, the SID Lys:NE estimated was 0.61 g/Mcal at 65 kg (the inflection point) and 0.48 g/Mcal at 105 kg, where pre-2010 and post-2010 growth curves differed by 7 days, which are greater than those estimated by the NRC (Figure 5). These differences may be attributed to improved genetic potential for lean growth and increased metabolic needs for Lys (Figure 1). According to Rostagno et al [77], the optimal SID Lys:NE (5.7 to 2.9 g/Mcal from 7 to 125 kg) for pigs raised between 2016 and 2023 were greater than those reported by the NRC [31], supporting the idea that optimal SID Lys:NE should be greater for pigs post-2010 compared with pigs prior to 2010 (Table 3; Figure 1). However, the optimal SID Lys:NE for ADG from 5 to 7 kg (5.8 g/Mcal in one-slope broken line model, 5.6 g/Mcal in quadratic model, and 6.1 g/Mcal in the quadratic broke line model) were not greater than the value reported by the NRC [31] (6.1 g/Mcal). A possible explanation may be the reduced birth weight of pigs born post-2010 (Figure 1) due to increased litter size of hyper-prolific sows, which could result in a lower SID Lys:NE from 5 to 7 kg.

Sex also plays an important role in determining optimal SID Lys:NE. Gilts typically exhibit a greater proportion of lean gain relative to fat gain compared with barrows, despite their slower growth and lower energy feed intake. The greater proportion of lean gain in gilts would increase their Lys requirement per unit of energy intake, resulting in a greater optimal SID Lys:NE for optimal growth performance in gilts [78]. This is supported by the higher SID Lys:NE for ADG estimated in gilts compared with barrows during the finishing phase, consistent with previous studies reporting that gilts have greater SID Lys:NE than barrows [78]. This suggests that different optimal SID Lys:NE values should be applied for barrows and gilts, particularly during the finishing phase.

Prediction equations for the optimal SID Lys:NE based on BW range of pigs were developed in this review to estimate the requirement for ADG based on 3 different models (Figure 4). G:F is a measure of growth relative to feed intake and is sensitive to AA imbalances or changes in Lys levels. However, prediction equations could not be established for G:F due to the lack of available optimal SID Lys:NE in the nursery phase (7 to 11 kg). Considering that G:F is calculated as weight gain divided by feed intake, it is influenced by changes in both variables. As pigs grow, changes in both weight gain and feed intake would alter G:F in unpredictable ways, making AA requirements for G:F difficult to estimate [79–81]. Therefore, prediction equations for the optimal SID Lys:NE were developed only for ADG, and not for G:F. Collectively, the optimal SID Lys:NE estimated in this review are higher than those recommended by the NRC [31], highlighting the need to update the SID Lys:NE to optimize growth performance of pigs.

## OPTIMAL STANDARDIZED ILEAL DIGESTIBLE METHIONINE TO STANDARDIZED ILEAL DIGESTIBLE LYSINE

Met, a sulfur-containing AA, is essential not only for protein deposition but also for the production of various biomolecules such as cytokines, glutathione, and acute phase proteins in pigs [82,83]. Met serves as a methyl donor via S-adenosylmethionine, which is required for DNA methylation and polyamine synthesis [84], and it can be converted to cysteine, an important building block for proteins and contributes to antioxidant capacity [83]. Considering its diverse biochemical roles, the use for Met is expected to be higher in pigs exposed to immune challenges than in non-challenged pigs [85].

Nursery pigs would require a greater dietary SID Met to SID Lys ratio (SID Met:Lys) compared with growing-finishing pigs due to their immature immune systems and greater susceptibility to immune challenges [85,86]. However, the results of the meta-analyses in this review do not support the assumption, which indicate that weaning stress alone does not increase the dietary SID Met:Lys unless nursery pigs are directly immune challenged [31,87].

The meta-analyses for optimal SID Met:Lys included 40 experiments (12 from 5 to 7 kg, 17 from 7 to 11 kg, and 11 from 11 to 25 kg; Table 4). The results indicate that the optimal SID Met:Lys for ADG remain relatively constant as BW of pigs increases, ranging from 0.25 to 0.27 in the one-slope broken line model and 0.32 to 0.37 in the quadratic model from 5 to 25 kg (Table 5). In contrast, the SID Met:Lys for ADG (0.37; standard error [SE] = 0.024) and G:F (0.40; SE = 0.020) in 11 to 25 kg are greater than those in 7 to 11 kg in the quadratic model (0.32; SE = 0.012 and 0.34; SE = 0.022, respectively). The SID Met:Lys for G:F is greater in 7 to 11 kg (0.27; SE = 0.019 and 0.35; SE = 0.033, respectively) compared with 5 to 7 kg (0.23; SE = 0.009 and 0.25; SE = 0.019, respectively) in the one-slope broken line model and the quadratic model. The optimal SID Met:Lys for ADG in the quadratic model (0.32 to 0.37) and quadratic broken line model (0.30 to 0.35) are generally greater than that reported by the NRC [31] (0.30; Table 3). The greater SID Met:Lys estimated compared with the NRC [31] is likely attributable to the improved genetic potential of modern pigs, which increases their Met requirement [88]. The optimal SID Met:Lys estimated by Rostagno et al [77] using data from pigs raised between 2016 to 2023 (0.33) as well as the value published by the CVB [89] in 2021 (0.33) is also greater than that reported by the NRC [31]. This indicates that the increased requirement for greater SID Met:Lys is not region-specific but rather a universal trend.

The optimal SID Met:Lys estimated are lower than those reported by Chae et al [87], ranging from 0.32 to 0.42. This difference may be explained by the exclusion of data from pigs exposed to immune challenges (*Escherichia coli* or unsanitary condition) in this review, whereas such data were included in the study by Chae et al [87]. In this review, the optimal SID Met:Lys for pigs over 25 kg were not provided because changes in SID Met:Lys did not affect the growth performance of pigs over 25 kg and thus, reliable estimates could not be obtained. This result is supported by previous studies reporting that growth performance was even reduced with increasing SID Met:Lys [90,91], likely due to Met toxicity [92,93]. Collectively, this review suggests that optimal SID Met:Lys in diets would increase under immune challenged conditions; however, weaning stress alone does not necessarily increase SID Met:Lys.

## OPTIMAL STANDARDIZED ILEAL DIGESTIBLE THREONINE TO STANDARDIZED ILEAL DIGESTIBLE LYSINE

Thr is the third-limiting AA in growing pigs fed cereal grain- and soybean meal-based diets and supplemental Thr is commonly added to pig diets [94]. Beyond its role in protein synthesis, Thr is a major component of mucin (accounting for 15% of mucin’s AA composition) which protects the intestinal lining [95,96] and supports the production of immunoglobulins [95,97,98]. According to the NRC [31], the SID Thr to SID Lys ratio (SID Thr:Lys) estimated to compensate for endogenous Thr losses in the intestinal tract (1.45) is greater than the ratio for protein deposition (0.53), highlighting the critical role of Thr in intestinal function and mucin production [99].

The meta-analyses for optimal SID Thr:Lys in this review included 50 experiments (6 from 7 to 11 kg, 13 from 11 to 25 kg, 9 from 25 to 50 kg, 9 from 50 to 75 kg, and 13 from 75 to 100 kg; Table 6). The optimal SID Thr:Lys for ADG increase as BW of pigs increases in the one-slope broken line model (0.50 to 0.63), quadratic model (0.61 to 0.68), and quadratic broken line model (0.57 to 0.69; Table 7). These results indicate that weaning stress does not necessarily increase SID Thr:Lys, but it increases as pigs grow. The optimal SID Thr:Lys for G:F increase as BW of pigs increases in the one-slope broken line model (0.57 to 0.64). In contrast, the optimal SID Thr:Lys for G:F in the quadratic model remain relatively constant (0.68 to 0.71) from 7 to 75 kg and increased to 0.75 (SE = 0.117) from 75 to 100 kg. In the quadratic broken line model, the optimal SID Thr:Lys for G:F remain constant (0.64 to 0.71) from 11 to 75 kg, but is lower than the ratio obtained from 7 to 11 kg (0.74; SE = 0.070) and 75 to 100 kg (0.79; SE = 0.136).

As pigs grow, their intestinal surface area increases [19,100], leading to greater number of goblet cells [101]. Considering that mucin is secreted by goblet cells and covers the intestinal surface, the maintenance requirement for Thr associated with mucin production gradually increases as pigs grow. Protein deposition in pigs reaches its maximum between 60 and 80 kg before declining [31], although the change in protein deposition remains relatively constant (120 to 150 g/day). Therefore, the optimal SID Thr:Lys increases as pigs grow because the maintenance requirement for Thr increases, whereas Thr needed for protein deposition remains relatively constant. This trend is consistent with NRC [31] and Hahn and Baker [102], both reporting an increase in the optimal SID Thr:Lys with increasing BW of pigs. Optimal SID Thr:Lys of pigs can be increased by feeding high-fiber diets or immune challenges. Mathai et al [103] reported that feeding soybean hulls to 25 to 50 kg pigs increased the optimal SID Thr:Lys for ADG from 0.66 to 0.71. Wellington et al [104] reported that feeding sugar beet pulp and wheat bran to 20-kg pigs increased the optimal SID Thr:Lys for protein deposition from 0.68 to 0.78. This is likely because dietary fiber provides physio-chemical stimulation, which increases endogenous AA losses including mucin [47,103]. Immune challenges can increase the optimal SID Thr:Lys by stimulating the production of mucin and immunoglobulins [105].

In the meta-analyses of SID Thr:Lys, 16 of 50 experiments were conducted post-2010, representing 32% of the total number of experiments included (3 from 7 to 11 kg, 4 from 11 to 25 kg, 5 from 25 to 50 kg, 2 from 50 to 75 kg, and 2 from 75 to 100 kg). The optimal SID Thr:Lys for ADG and G:F estimated in this review are greater than those reported by NRC [31] (0.59 to 0.63). The optimal SID Thr:Lys estimated by Rostagno et al [77] using data from pigs raised between 2016 to 2023 (0.68) as well as the value published by the CVB [89] (0.65 to 0.68) is greater than that reported by the NRC [31] (0.58 to 0.66; Table 3). Collectively, this review confirms that increasing BW increases the optimal SID Thr:Lys due to a greater proportion of maintenance requirements in later growth phases, suggesting that applying different SID Thr:Lys values at different BW could optimize growth performance in pigs.

## OPTIMAL STANDARDIZED ILEAL DIGESTIBLE TRYPTOPHAN TO STANDARDIZED ILEAL DIGESTIBLE LYSINE RATIO

Trp is an aromatic AA with functions in the nervous, immune, and intestinal systems beyond protein synthesis. As the precursor of serotonin and melatonin, Trp regulates hypothalamic satiety signaling, stress responsiveness, and behavior, thereby affecting feed intake and growth [106–108]. Specifically, Trp can mitigate weaning stress or immune challenges through the production of serotonin in the brain, which can regulate cortisol levels and enhance antioxidant and anti-inflammatory capacity [109–113]. From this perspective, nursery pigs would require a greater dietary SID Trp to SID Lys ratio (SID Trp:Lys) compared with growing-finishing pigs because of their immature immune system, the negative impacts of weaning stress, and higher metabolic costs associated with immune activation [114]. However, the meta-analyses in this review indicate that weaning stress alone does not necessarily increase optimal SID Trp:Lys, and that optimal SID Trp:Lys increases primarily when pigs are directly exposed to immune challenges [82,115].

The meta-analyses for optimal SID Trp:Lys included 71 experiments (15 from 7 to 11 kg, 25 from 11 to 25 kg, 12 from 25 to 50 kg, 5 from 50 to 75 kg, 8 from 75 to 100 kg, and 6 from 100 to 135 kg; Table 8). The results indicate that the optimal SID Trp:Lys for ADG remain relatively constant as BW of pigs increases, ranging from 0.16 to 0.20 in the one-slope broken line model, 0.20 to 0.25 in the quadratic model, and 0.20 to 0.24 in the quadratic broken line model (Table 9). For G:F, the optimal SID Trp:Lys remain constant as BW of pigs increases, ranging from 0.16 to 0.20 in the one-slope broken line analysis. In contrast, the optimal SID Trp:Lys decreases as BW of pigs increases based on ADG in the quadratic model (0.25 to 0.20) and G:F in the quadratic model (0.29 to 0.20) and quadratic broken line model (0.25 to 0.18). This result is consistent with previous studies that reported the optimal SID Trp:Lys was greater than 0.20 [116,117]. Interestingly, the optimal SID Trp:Lys estimated are greater than the ratios (0.16 to 0.18) reported by the NRC [31], which may indicate that the optimal Trp:Lys had been underestimated in the past [88]. A previous meta-analysis [118] conducted pre-2010 reported that the optimal SID Trp:Lys for maximizing growth of pigs ranged from 0.17 to 0.22. Additionally, Rostagno et al [77] showed that pigs used in studies from 2016 to 2023 had a SID Trp:Lys (0.20 to 0.21), which was greater than that (0.18) reported by the NRC [31] (Table 3). The CVB [89] also reported an optimal SID Trp:Lys was 0.20, which is greater than the ratio reported by the NRC [31].

In the meta-analyses on SID Trp:Lys, 49 of 71 experiments were conducted post-2010, representing 69% of the total number of experiments included (9 from 7 to 11 kg, 17 from 11 to 25 kg, 10 from 25 to 50 kg, 3 from 50 to 75 kg, 6 from 75 to 100 kg, and 4 from 100 to 135 kg). In a previous study, the optimal SID Trp:Lys was greater in pigs raised under unsanitary conditions (0.21) compared with those raised under normal conditions (0.20), likely because Trp helps reduce inflammatory responses in the intestinal tract by lowering acute phase protein production [119]. A greater SID Trp:Lys (0.24) mitigated the negative impact of *Escherichia coli* challenge on growth performance of nursery pigs compared with a lower SID Trp:Lys (0.16) [82]. Therefore, the optimal SID Trp:Lys was expected to be lower than that reported in a previous meta-analysis that included data from immune-challenged pigs [87]. However, the estimated ratios in this review were not consistently lower, indicating that immune challenges may not uniformly increase the optimal SID Trp:Lys. Collectively, this review suggests a greater optimal SID Trp:Lys (around 0.22) compared with those reported by the previous references [77,89,118] would be required to optimize growth performance in pigs.

## OPTIMAL STANDARDIZED ILEAL DIGESTIBLE VALINE TO STANDARDIZED ILEAL DIGESTIBLE LYSINE RATIO

Feed-grade L-Val has become commercially available since the mid-2000s, enabling lower dietary protein levels and preventing branched-chain amino acid (BCAA) imbalances in pig diets [75,120]. Beyond protein synthesis and muscle deposition, Val supports immune cell function and serves as a key metabolic substrate in rapidly dividing tissues [121,122]. According to the NRC [31], the SID Val to SID Lys ratio (SID Val:Lys) is 0.65 for all phases (Table 3). However, previous studies indicated that greater ratios (0.68 to 0.70) may be required to maximize growth or feed efficiency of nursery pigs [123–125].

The meta-analyses for SID Val:Lys in this review included 29 experiments (7 from 7 to 11 kg, 16 from 11 to 25 kg, and 6 from 25 to 50 kg; Table 10). Among these, 24 experiments were conducted post-2010, representing 83% of the total number of experiments included (5 from 7 to 11 kg, 14 from 11 to 25 kg, and 5 from 25 to 50 kg). The optimal SID Val:Lys for ADG from 7 to 11 kg (0.70; SE = 0.022) and 11 to 25 kg (0.67; SE = 0.014) are greater than that from 25 to 50 kg (0.64; SE = 0.014) in the one-slope broken line model (Table 11). The optimal SID Val:Lys for ADG from 11 to 25 kg (0.70; SE = 0.007) is greater than that from 25 to 50 kg (0.68; SE = 0.015) in the quadratic model. However, the optimal SID Val:Lys for ADG in the quadratic broken line model remain consistent from 11 to 50 kg. The greater SID Val:Lys estimated aligns with the ratios estimated by the CVB [89] (0.67) and Rostagno et al [77] (0.69 to 0.70).

In nursery pigs, weaning stress compromises intestinal morphology and immune function, thereby increasing maintenance needs for tissue repair and immune activity [76], which may explain the greater SID Val:Lys for ADG from 7 to 11 kg in the one-slope broken line model and quadratic broken line model. In growing-finishing pigs, high dietary leucine (Leu) levels elevate the optimal SID Val:Lys. In growing-finishing pig diets, the inclusion rate of corn by-products (e.g., corn distillers dried grains with solubles or corn gluten feed) is typically greater compared with nursery pig diets. These feedstuffs contain high levels of Leu, which enhance BCAA catabolism and can lead to BCAA antagonism [126,127]. Specifically, high dietary Leu content activates BCAA catabolic pathways (branched-chain aminotransferase and branched-chain α-keto acid dehydrogenase complex) and exacerbates BCAA antagonism, thereby enhancing Val degradation and elevating SID Val:Lys.

In summary, greater dietary SID Val:Lys would be needed in nursery phases to mitigate weaning stress and in growing-finishing phases to offset excess dietary Leu compared with the ratio provided by the NRC [31]. However, this review was unable to estimate the optimal SID Val:Lys for growing-finishing pigs, possibly due to the limited number of available experiments.

## OPTIMAL STANDARDIZED ILEAL DIGESTIBLE ISOLEUCINE TO STANDARDIZED ILEAL DIGESTIBLE LYSINE RATIO

Ile is another BCAA and the NRC [31] recommends SID Ile to SID Lys ratio (SID Ile:Lys) of 0.50 to 0.54. However, previous studies suggested that greater ratios (0.55 to 0.59) are needed to maximize growth and feed efficiency of nursery pigs [128,129]. The greater SID Ile:Lys estimated in the previous studies compared with the NRC [31] is partially attributed to feedstuffs used in the diets. When estimating Ile requirements, blood co-products such as blood cells are commonly included in diets because they contain low contents of Ile. In contrast to Ile, blood cells are rich in Leu, resulting in diets containing blood cells with high Leu contents [128,129]. Therefore, SID Ile:Lys estimated using diets containing blood cells might be overestimated due to BCAA imbalance caused by their high Leu content [130]. In contrast, SID Ile:Lys estimated using diets without blood cells (0.54) was lower compared with diets containing blood cells (0.59), indicating that the estimation of SID Ile:Lys would be dependent on dietary Leu content.

However, because SID Ile:Lys estimated from low-Leu diets still exceeds the ratio provided by the NRC [31], pigs likely require more Ile due to their improved genetic potential compared with the past.

Rostagno et al [77] estimated SID Ile:Lys to be 0.55, supporting the greater SID Ile:Lys estimated in this review compared with the NRC [31] (Table 3). In the meta-analyses on SID Ile:Lys, 11 of 19 experiments were conducted post-2010, representing 58% of the total number of experiments included (3 from 7 to 11 kg, 5 from 11 to 25 kg, and 3 from 25 to 50 kg). The meta-analyses for SID Ile:Lys included 19 experiments (5 from 7 to 11 kg, 10 from 11 to 25 kg, and 4 from 25 to 50 kg; Table 12). This review was unable to estimate the optimal SID Ile:Lys for pigs over 50 kg.

The optimal SID Ile:Lys for ADG remain constant as BW of pigs increases, ranging from 0.48 to 0.51 in the one-slope broken line model, 0.57 to 0.60 in the quadratic model, and 0.57 to 0.61 in the quadratic broken line model (Table 13). The optimal SID Ile:Lys for G:F from 7 to 11 kg (0.40; SE = 0.0003 and 0.41; SE = 0.010) is lower than that from 11 to 25 kg (0.46; SE = 0.014 and 0.52; SE = 0.050) and 25 to 50 kg (0.46; SE = 0.002 and 0.58; SE = 0.100) in the one-slope broken line model and quadratic broken line model. These results indicate that weaning stress does not necessarily increase Ile requirements.

## SUMMARY

Meta-analyses from this review indicate that the optimal SID Lys:NE for ADG (5.8 to 2.9 g/Mcal from 7 to 135 kg) are greater than those reported by the NRC [31]. Increasing SID Lys:NE increases daily requirements (g/d) of the other AAs because their supply is expressed as a ratio relative to Lys. Additionally, the optimal SID AA:Lys estimated exceed the values reported by the NRC [31]. The SID Lys:NE and SID AA:Lys estimated are comparable to those of Rostagno et al [77] and CVB [89], indicating that the need to increase AA requirements is a general trend, rather than being region-specific. Collectively, this review implies that pigs with improved genetic potential have greater AA requirements (SID Lys:NE and SID AA:Lys) than in the past. The optimal SID Met:Lys (0.33), SID Trp:Lys (0.22), and SID Ile:Lys (0.59) for ADG estimated remain constant with increasing BW of pigs. In contrast, the optimal SID Thr:Lys for ADG (0.59 to 0.69) increases from 7 to 100 kg, which may be due to an increased maintenance requirement for Thr. The optimal SID Val:Lys for ADG is estimated at 0.75 in nursery pigs, which is greater than 0.70 estimated in growing pigs, likely reflecting its role in mitigating weaning stress. Therefore, SID Thr:Lys and SID Val:Lys ratios should be adjusted in pig diets according to BW.

## CONCLUSION

The optimal dietary SID Lys:NE and SID AA:Lys for ADG are greater than the ratios provided by the NRC, and different ratios of SID Thr:Lys and SID Val:Lys should be applied for pigs with different BW.

## Figures and Tables

**Table 1:** Growth performance of pigs in response to the standardized ileal digestible (SID) lysine (Lys) to net energy (NE) ratio (SID Lys:NE, g/Mcal)

Item	n	Average BW (kg)		SID Lys:NE (g/Mcal)			NE (kcal/kg)			Range of growth performance		
			
		IBW	FBW	Mean	Min	Max	Mean	Min	Max	ADG (g/d)	ADFI (g/d)	G:F
BW range												
5 to 7 kg	4	6.2	7.9	5.42	3.86	6.95	2,528	2,472	2,593	221 to 298	244 to 319	0.79 to 1.13
7 to 11 kg	25	7.5	12.5	5.19	2.54	6.95	2,474	2,183	2,597	175 to 509	235 to 773	0.42 to 0.93
11 to 25 kg	30	11.4	20.4	4.79	2.14	6.95	2,472	2,183	2,634	441 to 771	567 to 1,390	0.37 to 0.84
25 to 50 kg	14	28.0	52.7	3.75	1.42	5.42	2,452	2,249	2,665	336 to 1,030	1,050 to 2,270	0.31 to 0.61
50 to 75 kg	11	46.5	77.2	3.32	1.69	5.29	2,472	2,342	2,650	572 to 1,200	1,447 to 3,612	0.28 to 0.52
75 to 100 kg	25	74.2	103.5	2.87	1.03	5.15	2,469	2,210	2,661	579 to 1,200	1,620 to 3,945	0.21 to 0.51
Barrows	7	75.6	102.9	2.43	1.03	5.09	2,518	2,342	2,661	590 to 1,200	2,278 to 3,945	0.21 to 0.43
Gilts	9	74.2	105.8	2.81	1.44	4.85	2,443	2,210	2,661	758 to 1,097	2,130 to 3,343	0.26 to 0.43
100 to 135 kg	9	99.1	125.5	2.53	1.61	3.84	2,575	2,478	2,675	721 to 1,080	2,331 to 3,590	0.25 to 0.36

BW, body weight; IBW, initial body weight; FBW, final body weight; ADG, average daily gain; ADFI, average daily feed intake; G:F, gain-to-feed ratio.

**Table 2:** Estimated standardized ileal digestible (SID) lysine (Lys) to net energy (NE) ratio (SID Lys:NE, g/Mcal) based on growth performance with 3 different statistical models

Item	One-slope broken line model			Quadratic model^1)^			Quadratic broken line model		
		
	SID Lys:NE	SE	p-value	SID Lys:NE	SE	p-value	SID Lys:NE	SE	p-value
Average daily gain									
5 to 7 kg	5.76	0.003	<0.001	5.58	0.254	<0.001	6.07	0.520	<0.001
7 to 11 kg	5.19	0.125	<0.001	5.82	0.203	<0.001	5.87	0.220	<0.001
11 to 25 kg	4.92	0.090	<0.001	5.68	0.201	<0.001	5.93	0.229	<0.001
25 to 50 kg	3.54	0.128	<0.001	4.23	0.210	<0.001	4.24	0.327	<0.001
50 to 75 kg	3.14	0.120	<0.001	4.02	0.245	<0.001	3.96	0.267	<0.001
75 to 100 kg	2.98	0.089	<0.001	3.57	0.088	<0.001	3.70	0.325	<0.001
Barrows	2.81	0.196	<0.001	3.65	0.336	<0.001	3.50	0.749	<0.001
Gilts	3.03	0.268	<0.001	3.79	0.382	<0.001	3.80	0.666	<0.001
100 to 135 kg	2.55	0.161	<0.001	2.77	0.130	<0.001	2.88	0.478	<0.001
Gain-to-feed ratio									
5 to 7 kg	5.76	0.370	<0.001	5.79	0.380	0.001	6.42	0.630	0.002
7 to 11 kg	5.59	0.004	<0.001	N/A	N/A	N/A	N/A	N/A	N/A
11 to 25 kg	5.43	0.097	<0.001	6.32	0.306	<0.001	6.67	0.304	<0.001
25 to 50 kg	4.20	0.187	<0.001	5.61	1.016	<0.001	5.91	1.069	<0.001
50 to 75 kg	3.54	0.128	<0.001	4.49	0.298	<0.001	4.63	0.360	<0.001
75 to 100 kg	3.13	0.003	<0.001	3.68	0.124	<0.001	4.04	0.252	<0.001
Barrows	2.98	0.221	<0.001	3.87	0.375	<0.001	4.12	0.548	<0.001
Gilts	3.03	0.171	<0.001	3.74	0.251	<0.001	3.67	0.488	<0.001
100 to 135 kg	2.29	0.146	<0.001	2.69	0.096	<0.001	2.70	0.307	<0.001

1) 95% of the upper asymptotic value for SID Lys:NE (g/Mcal).

SE, standard error; N/A, not available.

**Table 3:** Comparison among the sources of the optimal standardized ileal digestible (SID) lysine (Lys) to net energy (NE) ratio (SID Lys:NE, g/Mcal) and SID amino acids (AA) to SID Lys ratio (SID AA:Lys) in pigs from weaning to market weight

Item	NRC [31]	CVB [89]	Rostagno et al [77]	Suggestion^1)^
SID Lys:NE (g/Mcal)	6.13 to 2.46	N/A	5.80 to 2.89	5.95 to 2.83^2)^
SID AA:Lys				
Lysine	1.00	1.00	1.00	1.00
Methionine (Met)	0.30	0.33	0.33	0.33^3)^
Met+cysteine	0.55 to 0.59^4)^	0.60 to 0.62^4)^	0.60	N/A
Threonine (Thr)	0.58 to 0.66^4)^	0.65 to 0.68^4)^	0.68	0.59 to 0.69^5)^
Tryptophan	0.18	0.20	0.21 (0.20)^6)^	0.22^3)^
Valine (Val)	0.63 to 0.67^4)^	0.67	0.70 (0.69)^7)^	0.70 (0.7^3)8)^
Isoleucine (Ile)	0.50 to 0.54^4)^	0.53	0.55	0.59^3)^
Leucine	1.00	1.00	1.02 (1.00)^7)^	N/A
Histidine	0.34	0.32	0.33	N/A
Phenylalanine	0.59	0.54	0.54	N/A
Phenylalanine+tyrosine	0.93	0.95	1.00	N/A

1) The body weight ranges used to determine the SID AA:Lys for Met, Thr, Trp, Val, and Ile were 5 to 25 kg, 7 to 100 kg, 7 to 135 kg, 7 to 50 kg, and 7 to 50 kg, respectively.

2) The range of optimal SID Lys:NE was determined as the average ratio from the quadratic model and quadratic broken line model based on average daily gain in each growth phase.

3) The optimal SID AA:Lys was determined as the average ratio from the quadratic model and quadratic broken line model based on average daily gain across growth phases.

4) The optimal SID AA:Lys increases with increasing weight of the nursery to finishing pigs.

5) The range of optimal SID Thr:Lys range was determined as the average ratio from the quadratic model and quadratic broken line model based on average daily gain in each growth phase.

6) The optimal SID tryptophan (Trp):Lys decreases after pigs reach 18 kg.

7) The optimal SID AA:Lys decreases after pigs reach 27 kg.

8) The optimal SID Val:Lys was determined as the average ratio from the quadratic model and quadratic broken line model based on average daily gain in nursery pigs (7 to 11 kg).

N/A, not available.

**Table 4:** Growth performance of pigs in response to the standardized ileal digestible (SID) methionine (Met) to SID lysine (Lys) ratio (SID Met:Lys)

Item	n	Average BW (kg)		SID Met:Lys			SID Lys (%)			Range of growth performance		
			
		IBW	FBW	Mean	Min	Max	Mean	Min	Max	ADG (g/d)	ADFI (g/d)	G:F
BW range												
5 to 7 kg	12	5.6	7.9	0.29	0.17	0.53	1.29	0.95	1.58	77 to 387	153 to 617	0.38 to 0.99
7 to 11 kg	17	7.7	13.1	0.30	0.18	0.42	1.23	0.95	1.65	167 to 538	305 to 898	0.46 to 0.90
11 to 25 kg	11	11.4	23.3	0.28	0.09	0.58	1.23	0.95	1.39	136 to 665	436 to 1,241	0.30 to 0.74

BW, body weight; IBW, initial body weight; FBW, final body weight; ADG, average daily gain; ADFI, average daily feed intake; G:F, gain-to-feed ratio.

**Table 5:** Estimated optimal standardized ileal digestible (SID) methionine (Met) to SID lysine (Lys) ratio (SID Met:Lys) based on growth performance with 3 different statistical models

Item	One-slope broken line model			Quadratic model^1)^			Quadratic broken line model		
		
	SID Met:Lys	SE	p-value	SID Met:Lys	SE	p-value	SID Met:Lys	SE	p-value
Average daily gain									
5 to 7 kg	0.25	0.011	<0.001	0.33	0.014	<0.001	0.30	0.026	<0.001
7 to 11 kg	0.27	0.016	<0.001	0.32	0.012	<0.001	0.32	0.069	<0.001
11 to 25 kg	0.26	0.011	<0.001	0.37	0.024	<0.001	0.35	0.028	<0.001
Gain-to-feed ratio									
5 to 7 kg	0.23	0.009	<0.001	0.38	0.021	<0.001	0.25	0.019	<0.001
7 to 11 kg	0.27	0.019	<0.001	0.34	0.022	<0.001	0.35	0.033	<0.001
11 to 25 kg	0.24	0.008	<0.001	0.40	0.020	<0.001	0.32	0.023	<0.001

1) 95% of the upper asymptotic value for SID Met:Lys.

SE, standard error.

**Table 6:** Growth performance of pigs in response to the standardized ileal digestible (SID) threonine (Thr) to SID lysine (Lys) ratio (SID Thr:Lys)

Item	n	Average BW (kg)		SID Thr:Lys			SID Lys (%)			Range of growth performance		
			
		IBW	FBW	Mean	Min	Max	Mean	Min	Max	ADG (g/d)	ADFI (g/d)	G:F
BW range												
7 to 11 kg	6	6.9	11.4	0.61	0.26	1.02	1.14	1.08	1.21	134 to 431	238 to 640	0.49 to 0.89
11 to 25 kg	13	11.3	20.7	0.55	0.34	0.78	1.09	0.90	1.37	301 to 736	644 to 1,080	0.34 to 0.77
25 to 50 kg	9	24.6	44.8	0.64	0.44	0.90	0.89	0.74	0.94	322 to 933	1,088 to 1,989	0.30 to 0.57
50 to 75 kg	9	43.5	69.8	0.60	0.34	0.78	0.71	0.65	1.08	520 to 1,022	1,677 to 3,040	0.25 to 0.47
75 to 100 kg	13	66.1	102.6	0.62	0.47	0.79	0.72	0.61	0.76	771 to 1,077	2,231 to 3,280	0.31 to 0.40

BW, body weight; IBW, initial body weight; FBW, final body weight; ADG, average daily gain; ADFI, average daily feed intake; G:F, gain-to-feed ratio.

**Table 7:** Estimated optimal standardized ileal digestible (SID) threonine (Thr) to SID lysine (Lys) ratio (SID Thr:Lys) based on growth performance with 3 different statistical models

Item	One-slope broken line model			Quadratic model^1)^			Quadratic broken line model		
		
	SID Thr:Lys	SE	p-value	SID Thr:Lys	SE	p-value	SID Thr:Lys	SE	p-value
Average daily gain									
7 to 11 kg	0.50	0.061	<0.001	0.61	0.035	<0.001	0.57	0.092	0.002
11 to 25 kg	0.57	0.018	<0.001	0.63	0.017	<0.001	0.65	0.028	<0.001
25 to 50 kg	0.58	0.025	<0.001	0.68	0.015	<0.001	0.67	0.053	<0.001
50 to 75 kg	0.60	0.044	<0.001	0.67	0.068	<0.001	0.68	0.046	<0.001
75 to 100 kg	0.63	0.020	<0.001	0.67	0.051	<0.001	0.69	0.075	<0.001
Gain-to-feed ratio									
7 to 11 kg	0.58	0.035	<0.001	0.71	0.026	<0.001	0.74	0.070	<0.001
11 to 25 kg	0.58	0.015	<0.001	0.68	0.038	<0.001	0.71	0.050	<0.001
25 to 50 kg	0.57	0.013	<0.001	0.69	0.015	<0.001	0.64	0.030	<0.001
50 to 75 kg	0.61	0.014	<0.001	0.71	0.029	<0.001	0.69	0.033	<0.001
75 to 100 kg	0.64	0.021	<0.001	0.75	0.117	<0.001	0.79	0.136	<0.001

1) 95% of the upper asymptotic value for SID Thr:Lys.

SE, standard error.

**Table 8:** Growth performance of pigs in response to the standardized ileal digestible (SID) tryptophan (Trp) to SID lysine (Lys) ratio (SID Trp:Lys)

Item	n	Average BW (kg)		SID Trp:Lys			SID Lys (%)			Range of growth performance		
			
		IBW	FBW	Mean	Min	Max	Mean	Min	Max	ADG (g/d)	ADFI (g/d)	G:F
BW range												
7 to 11 kg	15	7.2	11.8	0.19	0.08	0.40	1.15	0.83	1.36	136 to 403	300 to 617	0.41 to 0.82
11 to 25 kg	25	12.7	24.0	0.19	0.09	0.42	1.00	0.82	1.35	216 to 807	407 to 1,512	0.22 to 0.79
25 to 50 kg	12	27.3	48.6	0.18	0.08	0.28	0.87	0.66	1.00	260 to 913	840 to 2,173	0.30 to 0.53
50 to 75 kg	5	50.1	81.4	0.18	0.07	0.25	0.75	0.65	0.83	479 to 1,060	1,733 to 3,230	0.27 to 0.41
75 to 100 kg	8	75.2	108.8	0.18	0.09	0.32	0.63	0.41	0.74	561 to 1,060	2,378 to 3,690	0.19 to 0.41
100 to 135 kg	6	91.9	126.4	0.18	0.12	0.25	0.65	0.51	0.85	759 to 1,160	2,261 to 3,630	0.26 to 0.38

BW, body weight; IBW, initial body weight; FBW, final body weight; ADG, average daily gain; ADFI, average daily feed intake; G:F, gain-to-feed ratio.

**Table 9:** Estimated optimal standardized ileal digestible (SID) tryptophan (Trp) to SID lysine (Lys) ratio (SID Trp:Lys) based on growth performance with 3 different statistical models

Item	One-slope broken line model			Quadratic model^1)^			Quadratic broken line model		
		
	SID Trp:Lys	SE	p-value	SID Trp:Lys	SE	p-value	SID Trp:Lys	SE	p-value
Average daily gain									
7 to 11 kg	0.17	0.004	<0.001	0.25	0.014	<0.001	0.22	0.011	<0.001
11 to 25 kg	0.20	0.024	<0.001	0.24	0.014	<0.001	0.23	0.029	<0.001
25 to 50 kg	0.16	0.003	<0.001	0.22	0.008	<0.001	0.22	0.001	<0.001
50 to 75 kg	0.18	0.011	<0.001	0.23	0.044	0.006	0.24	0.039	0.003
75 to 100 kg	0.17	0.010	<0.001	0.22	0.013	<0.001	0.22	0.025	<0.001
100 to 135 kg	0.17	0.013	<0.001	0.20	0.009	<0.001	0.20	0.008	<0.001
Gain-to-feed ratio									
7 to 11 kg	0.20	0.0003	<0.001	0.28	0.023	<0.001	0.25	0.022	<0.001
11 to 25 kg	0.17	0.007	<0.001	0.29	0.036	<0.001	0.19	0.020	<0.001
25 to 50 kg	0.17	0.001	<0.001	0.22	0.008	<0.001	0.20	0.011	<0.001
50 to 75 kg	0.17	0.013	<0.001	0.20	0.018	<0.001	0.21	0.026	0.001
75 to 100 kg	0.20	0.016	<0.001	0.24	0.022	<0.001	0.24	0.032	<0.001
100 to 135 kg	0.16	0.007	<0.001	0.20	0.013	<0.001	0.18	0.014	<0.001

1) 95% of the upper asymptotic value for SID Trp:Lys.

SE, standard error.

**Table 10:** Growth performance of pigs in response to the standardized ileal digestible (SID) valine (Val) to SID lysine (Lys) ratio (SID Val:Lys)

Item	n	Average BW (kg)		SID Val:Lys			SID Lys (%)			Range of growth performance		
			
		IBW	FBW	Mean	Min	Max	Mean	Min	Max	ADG (g/d)	ADFI (g/d)	G:F
BW range												
7 to 11 kg	7	7.8	12.4	0.65	0.49	0.85	1.17	0.94	1.43	184 to 457	304 to 639	0.57 to 0.81
11 to 25 kg	16	10.9	21.8	0.68	0.45	0.95	1.06	0.92	1.31	264 to 731	396 to 1,434	0.46 to 0.75
25 to 50 kg	6	27.5	43.1	0.67	0.55	0.80	0.97	0.90	1.10	621 to 1,020	1,315 to 2,230	0.39 to 0.58

BW, body weight; IBW, initial body weight; FBW, final body weight; ADG, average daily gain; ADFI, average daily feed intake; G:F, gain-to-feed ratio.

**Table 11:** Estimated optimal standardized ileal digestible (SID) valine (Val) to SID lysine (Lys) ratio (SID Val:Lys) based on growth performance with 3 different statistical models

Item	One-slope broken line model			Quadratic model^1)^			Quadratic broken line model		
		
	SID Val:Lys	SE	p-value	SID Val:Lys	SE	p-value	SID Val:Lys	SE	p-value
Average daily gain									
7 to 11 kg	0.70	0.022	<0.001	0.70	0.016	<0.001	0.76	0.050	<0.001
11 to 25 kg	0.67	0.014	<0.001	0.70	0.007	<0.001	0.70	0.015	<0.001
25 to 50 kg	0.64	0.014	<0.001	0.68	0.015	<0.001	0.71	0.031	<0.001
Gain-to-feed ratio									
7 to 11 kg	0.64	0.015	<0.001	0.69	0.017	<0.001	0.70	0.032	<0.001
11 to 25 kg	0.62	0.013	<0.001	0.73	0.023	<0.001	0.66	0.027	<0.001
25 to 50 kg	0.69	0.016	<0.001	0.71	0.024	<0.001	0.74	0.026	<0.001

1) 95% of the upper asymptotic value for SID Val:Lys.

SE, standard error.

**Table 12:** Growth performance of pigs in response to the standardized ileal digestible (SID) isoleucine (Ile) to SID lysine (Lys) ratio (SID Ile:Lys)

Item	n	Average BW (kg)		SID Ile:Lys			SID Lys (%)			Range of growth performance		
			
		IBW	FBW	Mean	Min	Max	Mean	Min	Max	ADG (g/d)	ADFI (g/d)	G:F
BW range												
7 to 11 kg	5	6.9	11.7	0.52	0.35	0.77	1.20	1.10	1.28	52 to 470	165 to 656	0.32 to 0.77
11 to 25 kg	10	10.9	21.8	0.53	0.33	0.72	1.00	0.75	1.21	173 to 608	298 to 1,030	0.39 to 0.72
25 to 50 kg	4	24.3	41.3	0.55	0.39	0.74	0.93	0.84	1.05	459 to 808	996 to 2,033	0.40 to 0.53

BW, body weight; IBW, initial body weight; FBW, final body weight; ADG, average daily gain; ADFI, average daily feed intake; G:F, gain-to-feed ratio.

**Table 13:** Estimated optimal standardized ileal digestible (SID) isoleucine (Ile) to SID lysine (Lys) ratio (SID Ile:Lys) based on growth performance with 3 different statistical models

Item	One-slope broken line model			Quadratic model^1)^			Quadratic broken line model		
		
	SID Ile:Lys	SE	p-value	SID Ile:Lys	SE	p-value	SID Ile:Lys	SE	p-value
Average daily gain									
7 to 11 kg	0.48	0.013	<0.001	0.57	0.017	<0.001	0.57	0.041	<0.001
11 to 25 kg	0.51	0.015	<0.001	0.58	0.022	<0.001	0.60	0.057	<0.001
25 to 50 kg	0.48	0.025	<0.001	0.60	0.032	<0.001	0.61	0.027	<0.001
Gain-to-feed ratio									
7 to 11 kg	0.40	0.0003	<0.001	0.71	0.175	0.015	0.41	0.010	<0.001
11 to 25 kg	0.46	0.014	<0.001	0.58	0.028	<0.001	0.52	0.050	<0.001
25 to 50 kg	0.46	0.002	<0.001	0.57	0.030	<0.001	0.58	0.100	<0.05

1) 95% of the upper asymptotic value for SID Ile:Lys.

SE, standard error.

## Data Availability

Upon reasonable request, the datasets of this study can be available from the corresponding author.
